# Optimizing jet pump efficiency via drag reducing polymers and enhanced efficiency definitions

**DOI:** 10.1038/s41598-024-54454-6

**Published:** 2024-02-15

**Authors:** Abdelsalam AlSarkhi, Abdulelah Kassar, Qasim Sahu, Rahul Gajbhiye

**Affiliations:** 1 Department of Mechanical Engineering and Center for Integrative Petroleum Research, King Fahd University of Petroleum and Minerals, Dhahran, Saudi Arabia; 2 Department of Petroleum Engineering and Center for Integrative Petroleum Research, King Fahd University of Petroleum and Minerals, Dhahran, Saudi Arabia; 3 Production Technology Division, EXPEC Advanced Research Center, Saudi Aramco Oil Company, Dhahran, Saudi Arabia

**Keywords:** Jet pumps, Air lift pump, Ejectors, Efficiency, Drag reducing polymers, Fossil fuels, Power distribution, Chemical engineering

## Abstract

Liquid jet pumps are widely used in various industrial applications for fluid mixing, circulation, and transport. The efficiency and performance of liquid jet pumps play a crucial role in determining their overall effectiveness and economic viability. The performance of liquid jet pumps is primarily affected by parameters such as motive fluid pressure, nozzle design, and entrainment ratio. Liquid jet pumps exhibit a notable drawback in terms of comparatively lower efficiency when compared to alternative pump types. The reduced overall efficiency of liquid jet pumps stems primarily from energy dissipation incurred during the entrainment process. To address this obstacle, a water-water loop system was implemented in conjunction with a liquid jet pump, followed by the introduction of drag-reducing polymers (DRPs) into the suction flow of the liquid jet pump using a specific configuration. This configuration led to a significant reduction in drag within the liquid jet pump, raising its efficiency in some cases from 13.8% to 26.7% with a drag reduction of 46%, subsequently improving its overall performance. The resulting enhancement was evaluated using various efficiency models documented in the existing literature to comprehensively assess the overall performance of the liquid jet pump. A new interpretation of jet pump efficiency has been shared, along with a comparison of the various efficiencies.

## Introduction

Jet ejectors are widely recognized for their simplicity, reliability, and cost-effectiveness, making them popular equipment for various industrial applications. However, their inherent inefficiency has spurred extensive research to identify and understand the key variables that influence their performance. This study examines previous experimental correlations that investigated the impact of different parameters on jet ejector efficiency. Several factors have been identified as crucial determinants of jet ejector performance, including Reynolds number, primary flow velocity, fluid temperature, throat characteristics, mixing chamber length, nozzle placement, molecular weight, and ratios of specific heat and pressure. Kumar et al.^1^ focused on nozzle placement (*NXP*) and found that the positioning of the nozzle significantly influenced the efficiency of the jet ejector. They recommended a nozzle placement between 0.5 and 1.0 of the throat diameter before entering the throat region for optimal merging of the primary and secondary streams. Holton and Schultz examined the effects of fluid temperature, while Work and Haedrich^2^ briefly studied the influence of fluid molecular weight. Furthermore, researchers have explored additional parameters to improve jet ejector efficiency. Hamedi-Estakhrsar et al.^3^ investigated the impact of divergence, convergence, length, and half-angles of the throat section on the ejector performance. Similarly, Kroll focused on nozzle placement, secondary fluid entrance, and primary flow velocity. Understanding the influence of these parameters on the jet ejector is essential for optimizing its performance in industrial processes. Through understanding how these factors interact, researchers and engineers can design and operate jet ejectors more effectively, thus enhancing their performance. To advance jet ejector technology, further research should be conducted to investigate and improve these findings. In the past, engineers relied on empirical methods, such as "rule-of-thumb" or "trial-and-error," to design jet ejectors. However, these methods often resulted in inefficient performance, requiring additional energy, materials, and effort. This study focuses on characterizing conventional jet ejectors based on the characteristics of the convergence region and classifies them into two categories: jet ejectors with constant area and jet ejectors with constant pressure. Recent research indicates that jet ejectors operating at constant pressure exhibit higher efficiency compared to those with a constant area. This efficiency advantage arises from the heightened efficiency of turbulent mixing within the jet ejector under a negative pressure gradient^4^. Notably, this effect is more pronounced in the constant-area jet ejector than in the constant-pressure jet ejector. Enhanced turbulent mixing, however, results in a reduction in ejector efficiency. Mathematical functions encompassing both categories of ejectors have been established by DeFrate and Hoerl^5^. These functions primarily focus on two key aspects: the relationship between the area ratio (*D*_*n*_/*D*_*t*_), where *D*_*n*_ and *D*_*t*_ are the diameters of the nozzle and the throat, respectively; the correlation between optimal motive and secondary stream velocity; as well as molecular weight and temperature.

In conventional jet pump applications, extensive literature has focused on optimizing performance through design modifications, emphasizing higher suction, improved fluid entrainment, and enhanced energy efficiency. While existing research explores design parameters and operational factors, there is a notable gap regarding alternative enhancements and operational considerations, such as the use of drag-reducing polymers (DRPs) for performance improvement without altering the pump's design. This study addresses this gap, comprehensively investigating the impact of DRPs on liquid jet pump performance under diverse operational conditions.

## Theory and definitions

### The usage of drag reducing polymers

Multiple techniques exist to prevent the occurrence of ejector pressure loss effects. The fluid's ability to flow is impeded by friction and pressure drops resulting from the interaction between eddies and the mixing chamber wall. Employing pumps is a straightforward method to elevate the fluid's pressure, which is crucial for reducing friction. However, the use of multiple pumps along the line is limited due to high energy consumption and associated costs. One of the most effective approaches is the implementation of drag-reducing agents as additives. Injecting a small amount of these chemicals into the stream increases the tunnel's capacity, reducing friction in the fluid and maintaining consistent tunnel conditions. In operational systems where pumping streams at high flow rates is necessary, leading to significant frictional pressure drops, drag-reducing agents can lower the power consumption required for pressurization, compensating for the pressure drops^6^.

Long-chain, high-molecular-weight polymers, such as polymethyl methacrylate (PMMA), polyethylene oxide (PEO), polyisobutylene (PIB), and polyacrylamide (PA), known as drag-reducing polymers (DRPs), have been extensively studied. Toms^6^ is credited with the formal discovery of drag reduction using polymers during experiments on polymer breakdown in pumps. Since then, numerous studies have been conducted to further comprehend and utilize this phenomenon for practical and fundamental purposes, resulting in a multitude of publications exploring single- and multiphase flows.

The use of DRPs has been shown to significantly increase the flow rate and reduce friction in oil–water two-phase flows. The Trans-Alaska pipeline serves as a notable real-world application where DRPs were employed on a large scale^7^. It has been observed that drag reduction primarily affects the buffer layer, while the viscous sublayer and log-law layer remain unaffected. DRPs increase the thickness of the buffer layer and diminish turbulence within it^8^.

While limited research has been conducted on drag-reducing agents for liquid–liquid flows^9^, available studies demonstrate that adding small amounts of polymers to the water phase can significantly increase the overall area of stratified flow and reduce pressure drop by approximately 65%. Furthermore, polymer additives have been found to alter the flow pattern map, liquid fraction, and interface height, as well as dampen interfacial waves^10^. Additionally, DRAs behavior is strongly influenced by Reynolds number^11^.

Due to the intense shear stresses encountered in pumps, the chemical structure of DRAs often does not withstand such conditions. As a result, DRAs need to be introduced after each pumping station. The effectiveness of DRAs is influenced by various factors, including viscosity, pipe diameter, flow velocity, and Reynolds number. Drag reduction is typically more effective in turbulent flows and increases as pipe diameter and flow viscosity decrease or as Reynolds number increases. Chang and Darby^12^ investigated the effects of shear degradation on polyacrylamide DRA solutions in 0.0046 m diameter pipes and found that DRAs experience aging, lose efficiency after a few days and degrade when subjected to shear. They also observed that freshly added DRA solutions exhibited lower friction factors compared to both the solvent alone and those exposed to pump shear.

Experimental research^13^ has been conducted to examine the impact of DRPs on oil–water flow patterns and pressure loss in a horizontal 1-inch acrylic pipe. The study revealed how molecular weights and polymer concentrations affect flow behavior and pressure reduction. The effectiveness of DRPs was also evaluated in relation to the salt content in the water phase, leading to the following observations:

The inclusion of DRPs decreases the level of turbulent mixing at higher mixture velocities, particularly in dispersed flow regimes, and can prevent the occurrence of the pressure gradient-induced phase inversion peak. The pressure gradient is significantly reduced, with the magnitude of the reduction depending on the molecular weight of the DRP, concentration, mixture velocity, and water percentage. In dispersed flow regimes at higher mixture velocities, the inclusion of DRPs minimizes the intensity of turbulent mixing and can eliminate the pressure gradient-induced phase inversion peak.

Although a few studies demonstrated DR in the laminar flow regime in curved pipes, the majority of drag reduction by additives occurs in the turbulent flow regime. Prior to a certain critical Reynolds number, the opposite is true; however, over this point, drag reduction by additives decreased with an increase in Reynolds number in the turbulent regime. This has been linked to the unequal dispersion of air bubbles or, depending on the situation, the breakdown of the polymer or surfactant^14^.

The efficiency of the drag-reducing polymer depends on the method used to introduce it into the flow. In addition to their concentration, the impact of DRAs on flow structure is dependent on concentration and molecular weight in addition to DR level. The DRA's elastic characteristics are linked to the concentration effect. The master polymer solution should be carefully prepared and introduced close to the surface film in a manner that spreads it evenly. High-shear pumps should be avoided during transfer into the pipe. The polymer's efficiency is quantified in terms of drag reduction (DR), calculated using the following formula:

1 $$DR= \frac{{\Delta P}_{without DRA}- {\Delta P}_{with DRA}}{{\Delta P}_{without DRA}}.$$

where: ${\Delta P}_{without DRA}$, ${\Delta P}_{with DRA}$ are the pressure drops without and with the drag reducing agent respectively.

### Jet pump efficiency definition

Efficiency plays a crucial role in assessing the performance of a liquid ejector, and numerous analytical models have been developed by corporate-sponsored academic institutions to optimize and enhance the design of liquid ejectors for optimal performance. One of the earliest models utilized a conceptual approach based on comparing the pressure drop of the suction flow to the discharge with the pressure drop of the motive flow to the discharge. This ratio was then multiplied by the entrainment ratio, which compares the flow rates of the suction and motive fluids. This conceptual model provided a foundation for subsequent models that followed a similar concept but incorporated analytical derivations based on the specific design characteristics of the liquid jet ejector. These analytical models focus on various aspects of the liquid ejector design, taking into account factors such as geometry, nozzle design, fluid properties, and operational parameters. By incorporating these variables into the models, engineers and researchers can gain valuable insights into the behavior and performance of liquid ejectors.

#### Impact of operational factors on ejector efficiency

Operational factors such as the fluid type and valve control also impact the ejector efficiency^15^. Additionally, an increased entrainment ratio has a substantial impact on the ejector's efficiency, highlighting the essential role the pump that powers the ejector plays. A new study thoroughly evaluated an electro pump’s efficiency at different flow rates^16^, the results showed that efficiency was at its peak at a particular flow rate, but that when the rate increased, efficiency showed a negative interaction. This emphasizes how crucial it is to determine the exact flow rate for the best possible system's efficiency, such as the ejector.

#### Impact of design factors on ejector efficiency

Experimental findings by Mellanby^17^ indicate that ejectors with rounded throats, such as cylindrical or conical with rounded entry, specifically 20°, create a larger maximum vacuum compared to those with progressive constriction tapering off to the outlet. Due to the jet throat entry having a typical angle of approximately 20°, it avoids causing undesirable shock and eddy losses at the convergence inlet. Interestingly, Mellanby also observed that the ability of a jet to entrain a fluid is not affected by the point along the jet where the entrained fluid enters. However, no specific explanations were provided regarding the potential causes of these observations.

The efficiency of an ejector is significantly influenced by the distance between the diffuser throat and the nozzle exit, as noted by Watson^18^. According to Watson, the maximum length of this distance decreases with increasing nozzle supply pressure and increases with rising vacuum. Furthermore, Watson emphasized the importance of positioning the nozzle concentrically with the throat's axis and highlighted that even minor adjustments in the throat size can have a significant impact on the amount of air entrained. If the throat is too small, choking can occur, causing the liquid to fill the throat and preventing the absorbed gas from passing through. Conversely, if the throat is too large, air leakage into the system can happen.

In terms of pressure recovery, a long and slowly diverging diffuser is preferred. Additionally, it has been demonstrated^19^ that a well-rounded entry provides the optimal shape for the entrance to the diffuser throat. This helps prevent unfavorable shock and eddy losses caused by the nozzle jet, which typically has an angle of approximately 20°. To mitigate these losses, it is recommended to have a conical or tapered entry with an angle greater than 20°.

In the diverging section, the angle of divergence (*θ*_*d*_) usually ranges from 4° to 10°; furthermore, abrupt deviations from this range should be avoided right after the throat because they could result in inadequate pressure recovery^4^. For optimal pressure recovery, a divergent length of approximately four to eight times the throat diameter is preferred. However, if necessary, this length can be reduced to as little as twice the throat diameter. Removing the divergence segment was found to result in a 20% reduction in the entrainment ratio (*Q*_*S*_/*Q*_*P*_).

#### Case 1: experimental efficiency

The experimental efficiency (*η*_*EX*_) is determined based on the pressure ratio. This efficiency calculation involves taking direct pressure readings obtained during the experiments and incorporating them into a well-established formula. This formula serves as a comprehensive and widely applicable approach where direct pressure and flow measurements are directly utilized. To calculate the experimental efficiency, precise pressure readings will be gathered from the experimental setup. These readings will be combined with the corresponding flow measurements obtained during the experiments. The experimental efficiency model is derived from the mathematical relationships presented below, which serve as the foundation for quantifying and analyzing the efficiency of the system:

2 $$M=\frac{{Q}_{s}}{{Q}_{p}} .$$

3 $$N=\frac{{P}_{d}{-P}_{s}}{{P}_{m}-{P}_{d}} .$$

4 $${\eta }_{EX}=M\times N .$$

where (*Q*_*s*_) and (*Q*_*p*_) are the secondary and primary flow rates, *P*_*m*_, *P*_*s*_, and *P*_*d*_ are the pressures for the primary, secondary, and discharge flow rates.

#### Case 2: analytical type 1 efficiency

An alternative efficiency formula we referred to as the Analytical type 1 efficiency (*η*_*A*1_), which was developed by ESDU 85032^20^, is worth considering. However, it is important to note that this formula is not universally applicable to all liquid–liquid ejectors due to several underlying assumptions. One significant assumption of this formula is that the nozzle outlet is located at the beginning of the mixing chamber, resulting in a (*NXP*) value of zero. The nozzle exit position (*NXP*) is the distance from the primary nozzle outlet to the intake of the mixing chamber. Upstream of the entrance to the mixing chamber, the *NXP* value exhibits a negative value when measured upstream, whereas it demonstrates a positive value when measured downstream^2^. By influencing both the entrainment ratio and the critical back pressure, primary *NXP* is a crucial geometrical component that significantly affects the ejector's efficiency. The critical back pressure and entrainment ratio are also affected by it. It has been demonstrated that ejector efficiency can vary depending on whether the nozzle exit position (*NXP*) is oriented inwardly or outwardly in the mixing chamber, influencing the entrainment and pressure lift ratio^3^. Additionally, it assumes that the mixing chamber has a constant area, which may not hold true in all cases, including our specific scenario. While the Analytical efficiency formula provides a useful framework for evaluating ejector performance, its limitations in accommodating variations in nozzle outlet positioning and mixing chamber geometry must be acknowledged. These assumptions may restrict the formula's accuracy and applicability to specific ejector configurations. As such, it is crucial to consider the specific design and operational characteristics of the liquid–liquid ejector under investigation when assessing its efficiency. Furthermore, the formula fails at a very low motive flow rate.

In our case, where the positioning of the nozzle outlet and the varying geometry of the mixing chamber are factors of interest, it becomes essential to explore alternative efficiency measures that account for these specific conditions. This allows for a more accurate evaluation of the ejector's performance and provides insights into its efficiency in converting the motive fluid's energy into useful work. By considering these factors and exploring alternative efficiency formulas tailored to our specific ejector design, we can obtain a more comprehensive understanding of its performance and make informed decisions regarding optimization and operational considerations. To proceed with the application of the equations, it is necessary to assign specific values to the parameters, which are commonly referred to as the loss coefficients. These coefficients, namely *K*_*p*_ (loss coefficient of the primary or motive flow), *K*_*s*_ (loss coefficient of the suction flow), *K*_*m*_ (loss coefficient of the mixing chamber), and *K*_*d*_ (loss coefficient of the diffuser or discharge), are assigned values of 0.7, 0.12, 1.36, and 0.43, respectively. These values are crucial for the analysis of the system. By utilizing the following equations, the analytical efficiency model (*η*_*A*1_) can be derived. These equations provide a systematic approach to obtaining and evaluating the efficiency of the system.

5 $${P}_{d}-{P}_{s}=\frac{1}{2}{\rho }_{p}{V}_{n}^{2}\left[2R+\frac{2C{M}^{2}{R}^{2}}{1-R}-{R}^{2}\left(1+{K}_{m}+{K}_{d}\right)\left(1+CM\right)\left(1+M\right)-C\left(1+{K}_{s}\right){\left(\frac{MR}{1-R}\right)}^{2}\right].$$

6 $${P}_{m}-{P}_{d}=\frac{1}{2}{\rho }_{p}{V}_{n}^{2}\left[-2R-\frac{2C{M}^{2}{R}^{2}}{1-R}+{R}^{2}\left(1+M\right)\left(1+CM\right)\left(1+{K}_{m}+{K}_{d}\right)+\left(1+{K}_{p}\right)\right].$$

7 $${\text{N}}=\frac{{P}_{d}{- P}_{s}}{{P}_{m}-{P}_{d}} .$$

8 $${\eta }_{A1}=M\times N .$$

where (*V*_*n*_) represents the velocity of the flow through the nozzle, while (*R*) denotes the area ratio between the mixing chamber area and the nozzle area, which has a specific value of 0.11169. Moreover, in our case, where both the motive and suction fluids are water, the variable (*C*) represents the density ratio between these fluids. The assigned value of (*C*) is 1, indicating that the density of the motive fluid is equal to the density of the suction fluid.

#### Case 3: analytical type 2 efficiency

To address the limitations of the assumptions made in the Analytical type 1 efficiency model (*η*_*A*1_), a new efficiency model, which we will refer to as the Analytical type 2 efficiency (*η*_*A*2_), was developed, as described in ESDU 93022^21^. The analytical type 2 efficiency takes into account the variable area of the mixing chamber, recognizing its crucial role and geometric characteristics that significantly impact the ejector's performance. It is worth noting that the analytical type 2 efficiency model assumes a nozzle exit position (*NXP*) of zero. While this assumption may slightly deviate the analytical type 2 efficiency from the actual efficiency observed in practice, our objective is to evaluate the performance and efficiency of the liquid ejector while considering the introduction of drag-reducing polymers (DRPs). Specifically, we will compare the efficiencies at two different motive flow rates, namely 1 and 1.45 GPM, using the analytical type 2 efficiency (*η*_*A*2_). By conducting this analysis, we aim to understand the impact of DRPs on the ejector's efficiency and identify any improvements or changes in its performance. The following equations represent the model.

9 $$ P_{{\text{d }}} - P_{{\text{s }}} = 0.5 \cdot \rho \cdot V_{n}^{2} \cdot \left[ {2 \cdot R + C \cdot M^{2} \cdot \left[ {\frac{{R \cdot A_{ME} }}{{1 - R \cdot A_{ME} }}} \right]^{2} \cdot \left[ {2 \cdot \left[ {\frac{{1 - R \cdot A_{ME} }}{{A_{ME} }}} \right] - \left( {1 + K_{s} } \right)} \right] - \left( T \right)} \right]. $$

10 $$ P_{{\text{m }}} - P_{{\text{d }}} = 0.5 \cdot \rho \cdot V_{n} ^{2} \cdot \left[ {1 + K_{p} - 2 \cdot R - 2 \cdot C \cdot M^{2} \cdot R \cdot R \cdot \left[ {\frac{{A_{ME} }}{{1 - R \cdot A_{ME} }}} \right] + \left( T \right)} \right]. $$

11 $$ T = \left( {X \cdot R^{2} \cdot \left( {1 + C \cdot M} \right) \cdot \left( {1 + M} \right)} \right) \cdot \left( {1 + K_{m} + K_{d} } \right). $$

12 $$ N = \left| {\frac{{P_{{\text{d }}} - P_{{\text{s }}} }}{{P_{{\text{m }}} - P_{{\text{d}}} }}} \right|. $$

13 $$ \eta_{A2} = M \times N. $$

Where (*A*_*ME*_) represents the area ratio between the mixing chamber inlet and outlet, in our case it is assigned with a 0.21 value. While (*X*) stands for the flow rate fraction between the discharge to the motive and suction flow rates (*Q*_*d*_/*Q*_*m*_ + *Q*_*s*_), in our case it equals 1. The remaining loss coefficients (*K*_*p*_*, K*_*s*_*, K*_*m*_, and *K*_*d*_) are assigned the values 0.7, 0.12, 1.36, and 0.43. The remaining parameters are assumed to be consistent with the previous models, meaning that they are kept unchanged and not subject to modification.

#### Case 4: head ratio efficiency

One of the developed formulas, referred to as the Head ratio efficiency (*η*_*HR*_), accounts for factors such as kinetic energy. Primary and secondary friction losses are attributable to the ejector design. By incorporating these factors into the head ratio efficiency model, a comprehensive assessment of the ejector's performance can be obtained^22^. Head ratio efficiency is a performance metric that assesses the effectiveness of a system in converting the available head into useful work or energy. Moreover, it provides insights into how efficiently the system utilizes the pressure energy of the motive fluid.

Frictional and pressure losses are common in liquid–liquid ejectors, which can impact their performance and efficiency. Frictional losses occur due to the resistance to flow caused by the interaction between the fluids and the walls of the ejector, or even due to the lack of optimal design of the ejector’s components. The loss coefficients are used to represent the magnitude of these losses and are used in the design and analysis of the ejector’s components. Motive, suction, discharge, and friction, which are referred to as *K*_*p*_, *K*_*s*_, and *K*_*d*_, are assigned the values of 0.7, 0.12, and 1.88, respectively. *K*_*fm*_, *K*_*fs*_, and *K*_*fd*_ are the friction loss coefficients for each component of the ejector, with values of 0.336, 0.37, and 0.56. By incorporating the coefficients of losses into the following equations:

14 $$N=\left|\frac{{H}_{d}-{H}_{s}}{{H}_{m}-{H}_{d}}\right|.$$

15 $${H}_{m}=\frac{{P}_{m}}{9810}+\left(1+{K}_{m}+{K}_{f,m}\right)\cdot \frac{{V}_{m}{ }^{2}}{2\cdot 9.81} .$$

16 $${H}_{s}=\frac{{P}_{s}}{9810}+\left(1+{K}_{s}+{K}_{f,s}\right)\cdot \frac{{V}_{s}{ }^{2}}{2\cdot 9.81} .$$

17 $${H}_{d}=\frac{{P}_{d}}{9810}+\left(1+{K}_{d}+{K}_{f,d}\right)\cdot \frac{{V}_{d}{ }^{2}}{2\cdot 9.81} .$$

18 $$M=\frac{{Q}_{s}}{{Q}_{m}} .$$

19 $${\eta }_{HR}=M\cdot N.$$

where *V*_*m*_, *V*_*s*_, and *V*_*d*_ are the velocities of motive, suction, and discharge. *Q*_*s*_ and *Q*_*m*_ are the flow rates of suction and motive. *P*_*m*_, *P*_*s*_, and *Pd* are the pressures for motive, suction, and discharge. The variable "M" denotes the entrainment ratio, specifically defined as the ratio of the suction flow rate (*Q*_*s*_) to the motive flow rate (*Q*_*m*_). This parameter quantifies the effectiveness of the propelling process within the system. On the other hand, the variable (*N*) signifies the pressure ratio of the ejector, which characterizes the relationship between the discharge pressure, the motive pressure, and the suction pressure.

## Experimental setup and procedure

### Flow loop description

**Figure 1:**
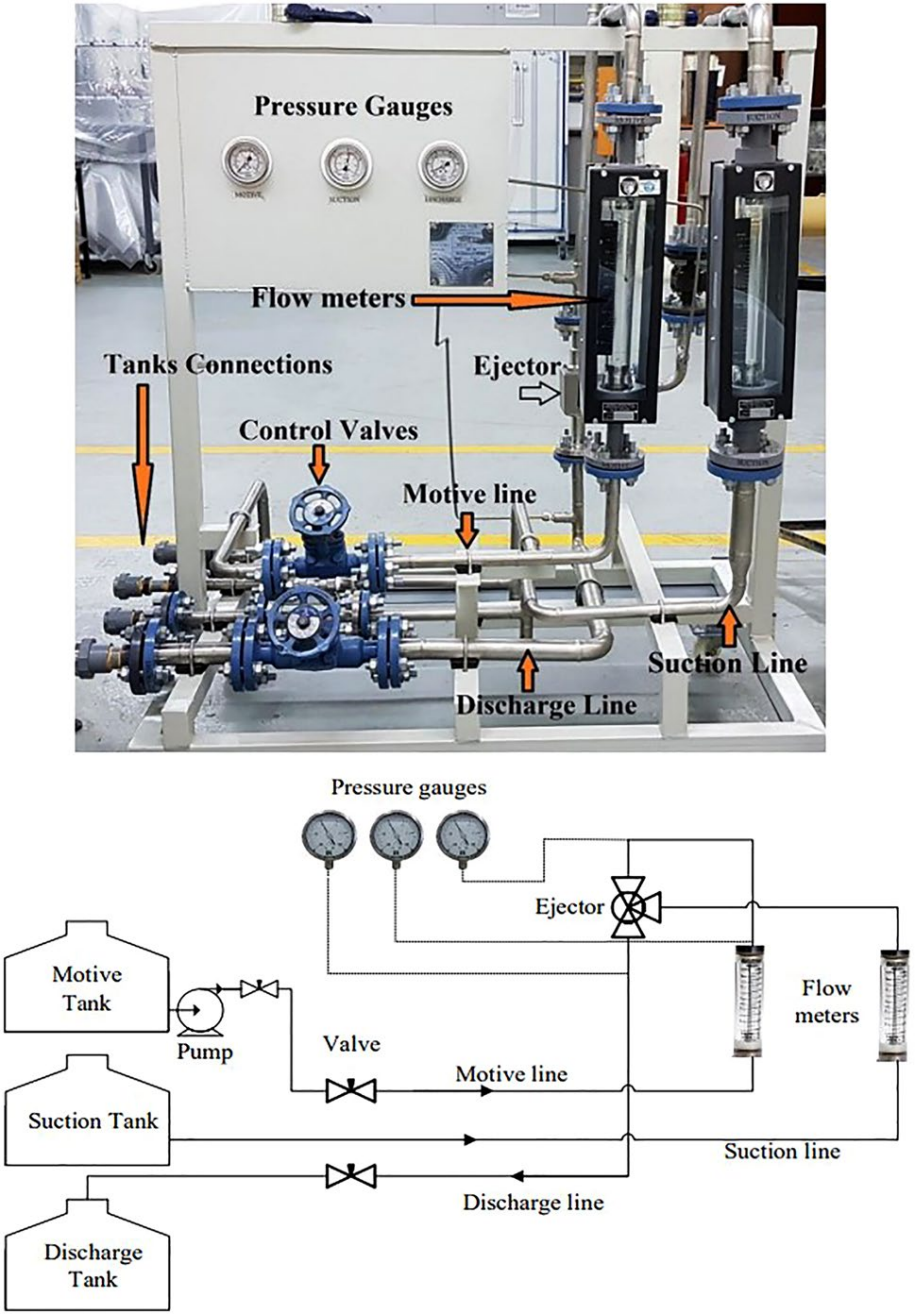
The experimental Flow Loop.

Figure 1 illustrates the experimental flow loop; it is designed for investigating the performance of a liquid–liquid ejector generating water-in-oil or oil-in-water emulsions. The flow loop comprises three main components: a motive liquid tank, a suction liquid tank, and a discharge tank. A valve connects the discharge tank to a water outlet aperture to make sure that the drag-reducing polymers concentration is constant. Conversely, when emulsion needs to be examined, the valve remains closed, enabling the tank to operate based on the specific experiment being conducted. To achieve the desired flow rates for the experiments, one centrifugal pump is integrated into the flow loop. The pump is equipped with a control valve, facilitating the regulation of the motive stream's flow rate. Additionally, flow meters and pressure gauges are installed along the motive and suction lines to measure the flow rate and pressure of the primary and secondary liquid streams. The liquid–liquid ejector functions by drawing its primary stream from the motive tank. Utilizing the pressure energy of the motive stream, the ejector effectively suctions the secondary stream from the suction tank and combines them to generate the desired emulsion. The resulting emulsion is then discharged through the ejector's discharge line. This experimental setup provides a controlled environment for studying the performance of the liquid–liquid ejector for emulsion generation. By precisely controlling the flow rates, monitoring pressure, and incorporating drag-reducing additives into the suction flow line to assess and study their influence on the performance of the ejector.

### Liquid ejector description

The jet pump is distinguished from other pump types primarily by its operating principle, wherein energy is transferred to the secondary fluid by utilizing the kinetic energy of the driving (primary) fluid rather than relying on mechanical components such as impellers or pistons. The main components of the ejector are the nozzle, mixing chamber, and diffuser.

### Ejector controlled operational modes

The experimental procedure for the suction-controlled mode encompassed a series of sequential steps to ensure accurate data collection and analysis. Initially, both the suction and motive tanks were filled with water to establish the necessary fluid volumes for the experiment. Next, the motive pump is activated while ensuring that all relevant valves are fully open to allow for unobstructed flow. To simulate varying suction flow rates, the suction flow rate control valve was gradually closed, thus reducing the influx of fluid from its maximum value to its minimum. Throughout this process, we meticulously recorded the corresponding flow rates and pressures for all interconnected lines within the experimental setup.

### Procedure

This section presents the outcomes of a parametric analysis, along with a comprehensive description of the procedure followed for each experiment. The analysis covers the impact of controlling the suction and discharge valves on the performance of the system. Furthermore, it explores the effect of drag-reducing polymers on the suction flow capacity, including tests conducted at various concentrations of these polymers to optimize the suction flow capacity at different motive flow rates. At the conclusion, a detailed discussion and analysis of the results are provided. The analysis delves into the findings and their implications, shedding light on the observed outcomes and relationships between the variables studied. The discussion incorporates a thorough examination of the experimental data and its relevance to the research objectives. This comprehensive analysis allows for a deeper understanding of the investigated phenomena and provides insights into the performance and potential enhancements of the ejector-based emulsion generation system.

#### Incorporating drag reducing polymers

In the same procedure in the suction-controlled mode experiment, an additional step was introduced to incorporate drag reduction polymers into the suction stream. This process involved mixing the polymers with 50 parts per million (PPM) and 100 (PPM) concentrations in a separate tank with a mixer equipped with a three-blade impeller. To ensure minimal degradation of the polymers, the mixing was conducted for a duration of one hour at a controlled speed of 85 revolutions per minute (RPM). ZETAG® 8165 (a copolymer of acrylamide and proprietary quaternized cationic monomer) was used as the water-soluble DRP. It is a high molecular weight copolymer and available as a free-flowing white powder. Table 1 lists the properties of ZETAG® 8165. Following the polymer mixing process, the experiments proceeded in a similar manner to the suction-controlled experiment. The data collection process began by measuring the flow rates and pressures within the system. Careful attention was given to maintaining the integrity and effectiveness of the polymers throughout the experiment. By adhering to the specified mixing time and speed and utilizing a dedicated tank, efforts were made to minimize any potential degradation or alteration of the polymers’ entanglement properties. Through these modified experimental steps, the impact of drag-reducing polymers on flow rates and pressures could be accurately evaluated, providing valuable insights into the effectiveness of these additives in improving the performance and efficiency of the system. Additionally, Table 2 illustrates the experimental parameters.

**Table 1:** Properties of water-soluble ZETAG®8165.

Properties	Description
Product name	Polyacrylamide (PAM)
Appearance	Off-white granular solid
Molecular weight	Very high
Bulk density	0.7 g cm^−3^
PH of 0.5% solution	Approx. 3.5
Solubility	Water-soluble

**Table 2:** Experimental parameters.

Parameter	Value
Water temperature	24 C^o^
Water density (*ρ*_*w*_)	997 kg/m^3^
Water dynamic viscosity (*µ*_*w*_)	0.0009096 Pa s
Nozzle diameter (*D*_*n*_)	2.7 mm
Area ratio (*R*)	0.11169
Density ratio (*C*)	1
Loss coefficients (*K*_*p*_, *K*_*d*_, *K*_*m*_, *K*_*d*_)	0.7, 0.12, 1.36, 0.43

## Results and discussion

**Figure 2:**
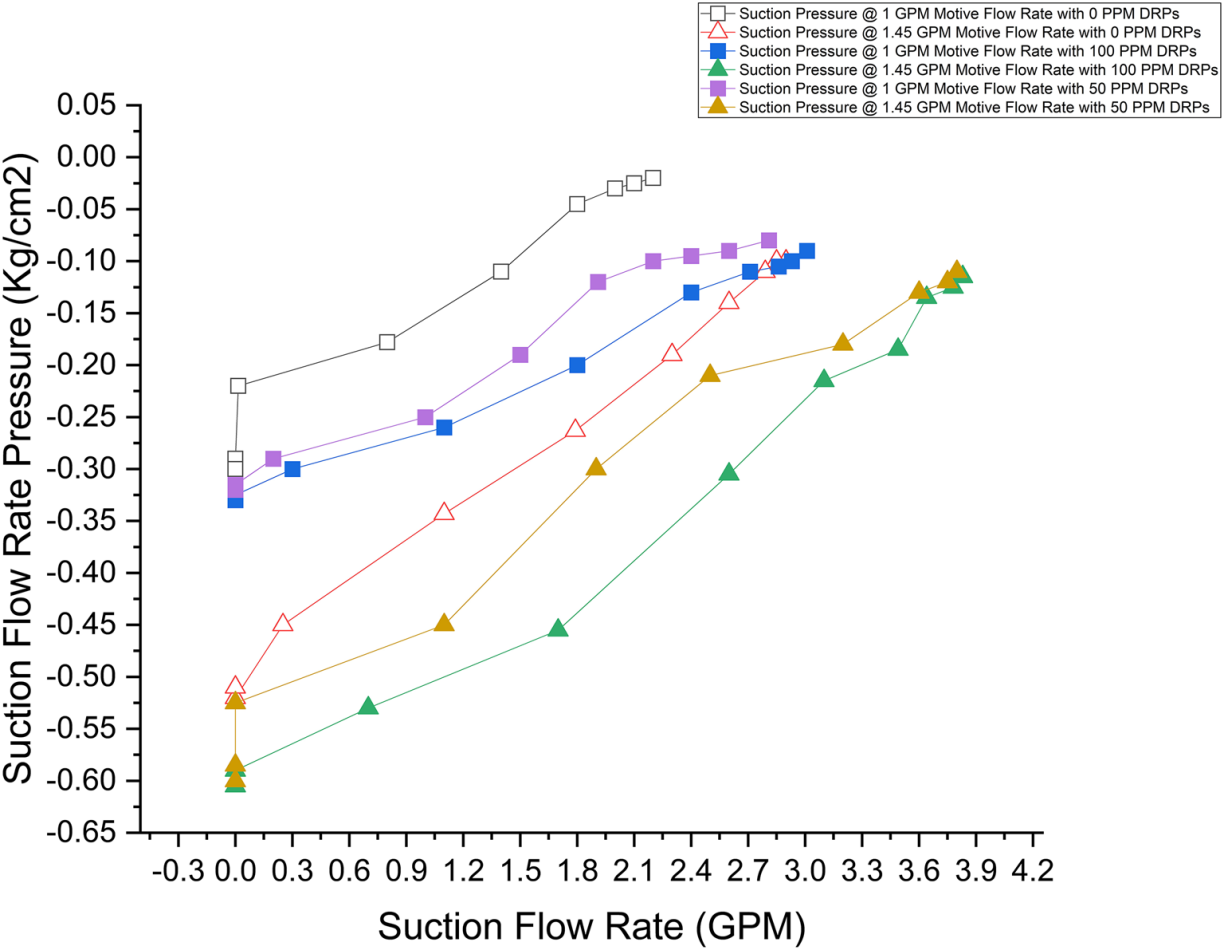
Variation of the suction flow rate pressure with the suction flow rate capacity at different motive flow rates and DRPs concentrations (100, 50 and 0 PPM).

The impact of drag-reducing polymers on suction flow rate pressure within the liquid jet was investigated. The suction flow rate decreases at different motive flow rates as the suction flow rate control valve is partially closed. This decrease in the suction flow rate is accompanied by a decrease in suction pressure, which drops from  − 0.1 kg/cm^2^ until it reaches  − 0.51 kg/cm^2^ at a 1.45 GPM motive flow rate and from 0.02 to  − 0.3 kg/cm^2^ at a 1 GPM motive flow rate. These changes in flow rate and pressure occur as the suction flow is controlled and adjusted through the partial closing of the suction control valve. Then drag-reducing polymers (DRPs) were introduced with 50 and 100 parts per million (ppm) concentrations within the suction line, and then the whole readings, such as suction flow rates and suction pressures, were taken again. Both experiments are presented in (Fig. 2) which represents the variation between the suction flow rate and pressure.

One notable observation is the significant increase in suction flow rate capacity with the addition of drag-reducing polymers (DRPs) at 50 ppm at the same motive flow rate. Specifically, when comparing the same motive flow rate, at 1.45 GPM motive flow rate, the suction flow rate capacity demonstrated a noteworthy increment of 0.9 gallons per minute (GPM). This increment translates to an increase of approximately 31% in the suction flow rate capacity; moreover, at 1 GPM motive flow rate, the suction flow rate increased to 2.81 GPM with a 28% increment in the suction flow rate capacity. It is worth noting that motive pressure remains unaffected by the presence of DRP. Additionally, there is a slight rise in suction pressure with the incorporation of DRP. Although the increase is relatively small, it indicates a change in the pressure dynamics within the system. On the other hand, the discharge pressure experiences a significant doubling effect. This is primarily due to the enhanced suction flow capacity resulting from the DRP addition and the reduction of the frictional pressure drop along the ejector. The increased suction flow rate ultimately leads to a higher discharge pressure. Furthermore, as the concentration of drag-reducing polymers increased to 100 ppm, the suction flow rate increased to 3.83 GPM at a motive flow rate of 1.45 GPM, slightly surpassing the flow rate observed at a concentration of 50 ppm. However, both concentrations (50 and 100 ppm) at a motive flow rate of 1.45 GPM exhibited nearly identical suction pressure levels. Tables 3 and 4 show the values of the pressures and flow rates at all suction valve capacities for motive flow rates of 1 and 1.45 GPM, respectively.

**Table 3:** Suction flow rates (SFR) and pressures (SP) comparison of the ejector at a motive flow rate of 1.0 GPM at different drag reducing polymers concentrations (0, 50 and 100 ppm) and different suction valve capacities (SVC).

Motive flow rate (GPM)	*SVC* (%)	*SFR* (GPM)			*SP* (kg/cm^2^)		
		0 (PPM)	50 (PPM)	100 (PPM)	0 (PPM)	50 (PPM)	100 (PPM)
1	100	2.2	2.81	3.01	− 0.02	− 0.08	− 0.08
1	90	2.2	2.6	2.93	− 0.02	− 0.09	− 0.09
1	80	2.1	2.4	2.86	− 0.03	− 0.1	− 0.1
1	70	2	2.2	2.71	− 0.03	− 0.1	− 0.1
1	60	1.8	1.91	2.4	− 0.05	− 0.12	− 0.12
1	50	1.4	1.5	1.8	− 0.11	− 0.19	− 0.19
1	40	0.8	1	1.1	− 0.18	− 0.25	− 0.25
1	30	0	0	0.3	− 0.22	− 0.29	− 0.29
1	20	0	0	0	− 0.29	− 0.32	− 0.32
1	10	0	0	0	− 0.3	− 0.32	− 0.32

**Table 4:** Suction flow rates (SFR) and pressures (SP) comparison of the ejector at a motive flow rate of 1.45 GPM at different drag reducing polymers concentrations (0, 50 and 100 ppm) and different suction valve capacities (SVC).

Motive Flow Rate (GPM)	*SVC* (%)	*SFR* (GPM)			*SP* (kg/cm^2^)		
		0 (PPM)	50 (PPM)	100 (PPM)	0 (PPM)	50 (PPM)	100 (PPM)
1.45	100	2.9	3.8	3.83	− 0.1	− 0.11	− 0.11
1.45	90	2.85	3.75	3.78	− 0.1	− 0.12	− 0.12
1.45	80	2.79	3.6	3.64	− 0.11	− 0.13	− 0.13
1.45	70	2.6	3.2	3.49	− 0.14	− 0.18	− 0.18
1.45	60	2.3	2.5	3.1	− 0.19	− 0.21	− 0.21
1.45	50	1.79	1.9	2.6	− 0.26	− 0.3	− 0.3
1.45	40	1.1	1.1	1.7	− 0.34	− 0.45	− 0.45
1.45	30	0.25	0	0.7	− 0.45	− 0.525	− 0.53
1.45	20	0	0	0	− 0.5	− 0.58	− 0.58
1.45	10	0	0	0	− 0.5	− 0.6	− 0.6

### Case 1

**Figure 3:**
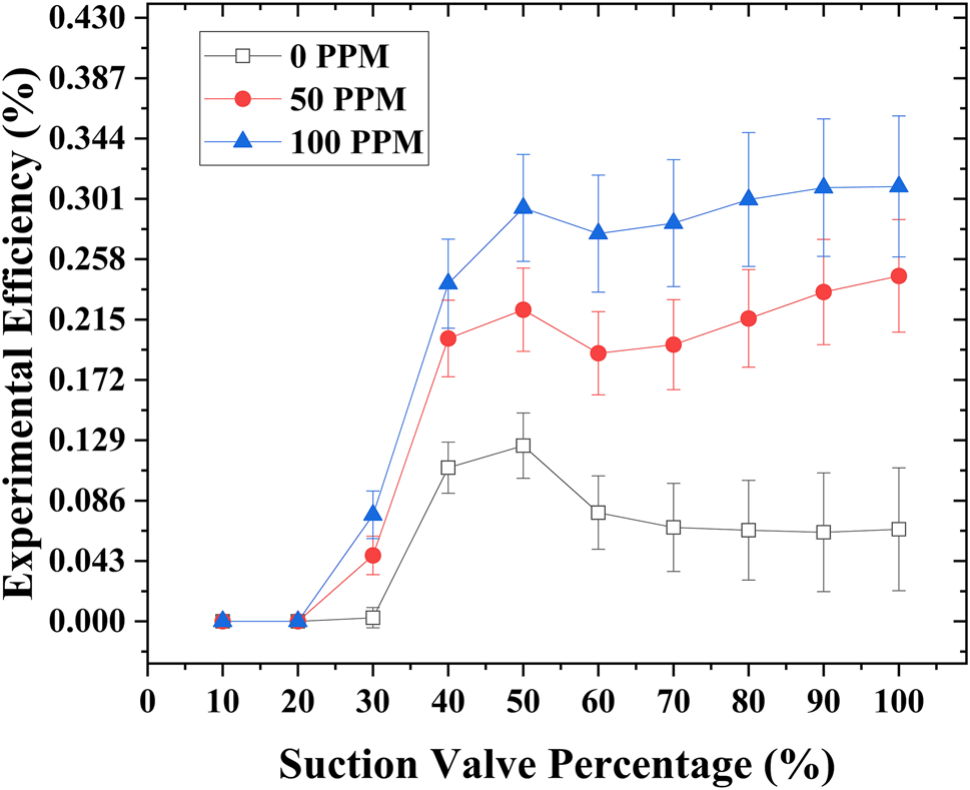
Experimental efficiency variation as a function of suction flow rate control valve capacity at a motive flow rate of 1 GPM, considering different concentrations of drag reducing polymers (DRPs) (0, 50, and 100 ppm).

The experimental efficiency showed a significant improvement through the addition of drag-reducing polymers (DRPs) at varying concentrations at a fixed motive flow rate of 1 gallon per minute (GPM). In the absence of DR/Ps, the experimental efficiency (*η*_*EX*_) was measured at 6.5% with a suction flow rate of 2.2 GPM when the suction flow rate control valve was fully opened. In addition, the highest efficiency of 12.5% was achieved with a 50% suction valve opening and a suction flow rate of 1.4 GPM. Subsequently, DRPs were introduced into the suction tank at a concentration of 50 ppm, resulting in a remarkable increase in the experimental efficiency (*η*_*EX*_). The efficiency increased from 6.5% to 22.7% with a suction flow rate of 2.81 GPM when the suction valve was fully opened. With a 50% suction flow rate control valve opening, the efficiency improved from 12.5% to 20.5% at a suction flow rate of 1.5 GPM. The concentration of DRPs was further increased to 100 ppm, leading to another substantial improvement in *η*_*EX*_. At a suction flow rate of 3.01 GPM, when the suction flow rate control valve was fully opened, the efficiency reached 31%. Similarly, with a 50% suction flow rate control valve opening, the efficiency stood at 29.45% with a suction flow rate of 1.8 GPM. Furthermore, it is worth noting that when the suction flow rate control valve is partially closed, an increase in efficiency was observed only in the absence of drag-reducing polymers (DRPs) at 40% and 50% opening of the valve, in comparison to when the valve was fully opened. However, when DRPs were introduced at different concentrations (50 and 100 ppm), closing the suction flow rate control valve only resulted in an efficiency increase at a 50% opening of the valve, as compared to a 60% opening. Nevertheless, even at a 50% opening of the suction flow rate control valve, the efficiency did not surpass that of the ejector when the valve was fully opened, as observed in the absence of DRPs. (Fig. 3) illustrates the relationship between suction flow rate control valve capacity and the experimental efficiency with varying concentrations of drag-reducing polymers (DRPs) (0, 50, and 100 ppm) at a fixed motive flow rate of 1 GPM.

**Fig.4:**
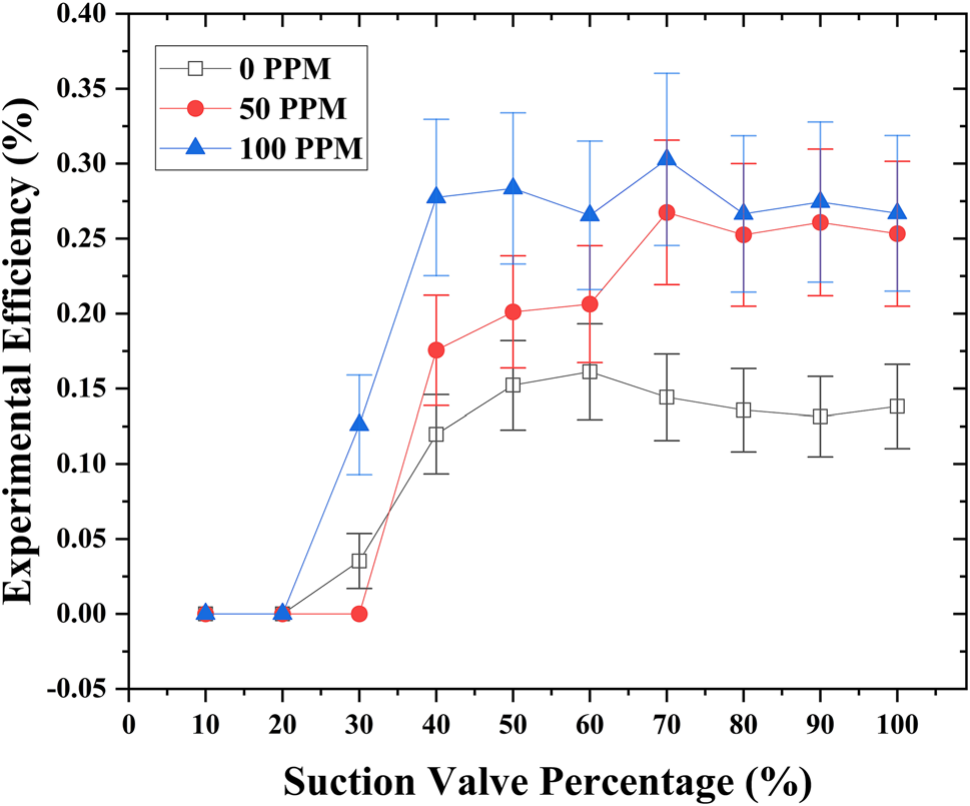
Relationship between the experimental efficiency and the suction flow rate control valve capacity for varying concentrations of drag reducing polymers (DRPs) (0 ppm, 50 ppm, and 100 ppm), at a fixed motive flow rate of 1.45 GPM.

When the motive flow rate increased to 1.45 GPM, a significant improvement was observed when drag-reducing polymers (DRPs) were introduced at different concentrations (50 and 100 ppm). When the concentration of DRPs was set at 50 ppm, an interesting enhancement in experimental efficiency was observed. Specifically, with the suction flow rate control valve fully opened, the experimental efficiency increased from 13.8% to 25.3%. This increase also resulted in an elevation of the suction flow rate from 2.9 to 3.8 GPM. Additionally, at a suction flow rate control valve opening of 70%, a slightly better experimental efficiency of 27% was obtained compared to the fully open position. Furthermore, when the concentration of DRPs was increased to 100 ppm, the experimental efficiency continued to improve. At the fully open suction flow rate control valve, the experimental efficiency increased to 26.7%, accompanied by a suction flow rate of 3.83 GPM. Interestingly, by reducing the suction flow rate control valve opening to 70%, the experimental efficiency further increased to 30.3% while maintaining a suction flow rate of 3.5 GPM. The observed increment in experimental efficiency during the closure of the suction flow rate control valve can be attributed to the decrease in suction pressure, which increases proportionally with the suction flow rate. Interestingly, when the suction valve closed by 50%, the efficiency was better compared to a 60% suction valve closure; however, the efficiency after 50% valve closure was drastically reduced, which contributed to entrainment and pressure ratios. At 50% closure, the flow rate reduced relatively less while the pressure significantly reduced; nevertheless, at 40% closure, the vacuum increased significantly, but due to the valve closure, insufficient flow was allowed to flow into the suction chamber. In the definition of efficiency, the ratio of the suction to the motive flow (*M*) has the most contribution to the efficiency formula, and a dramatic decrease in it leads to a dramatic reduction in the efficiency. This relationship suggests that controlling the suction flow rate through the valve can have a significant impact on the overall efficiency of the system, especially when combined with the introduction of drag-reducing polymers. (Fig. 4) depicts the graphical representation of the relationship.

Furthermore, for a better understanding of the phenomenon at both 50% and 70% closure of the suction valve, the equation of experimental model (2) should be written in the form of velocities:

20 $$M=\frac{{Q}_{s}}{{Q}_{M}} =\frac{{A}_{s}{V}_{s}}{{A}_{N}{V}_{N}} .$$

where, *A*_*s*_, and *A*_*N*_ are the areas for the suction pipe and motive nozzle. Closing the suction valve reduces the suction area, causing an increase in suction velocity and a slight decrease in flow rate due to increased flow resistance. In Eq. (3), both motive and discharge exhibit slight changes, while suction pressure undergoes a significant alteration. Furthermore, a slight decrease in suction pressure results in an increased pressure ratio (*N*).

**Figure 5:**
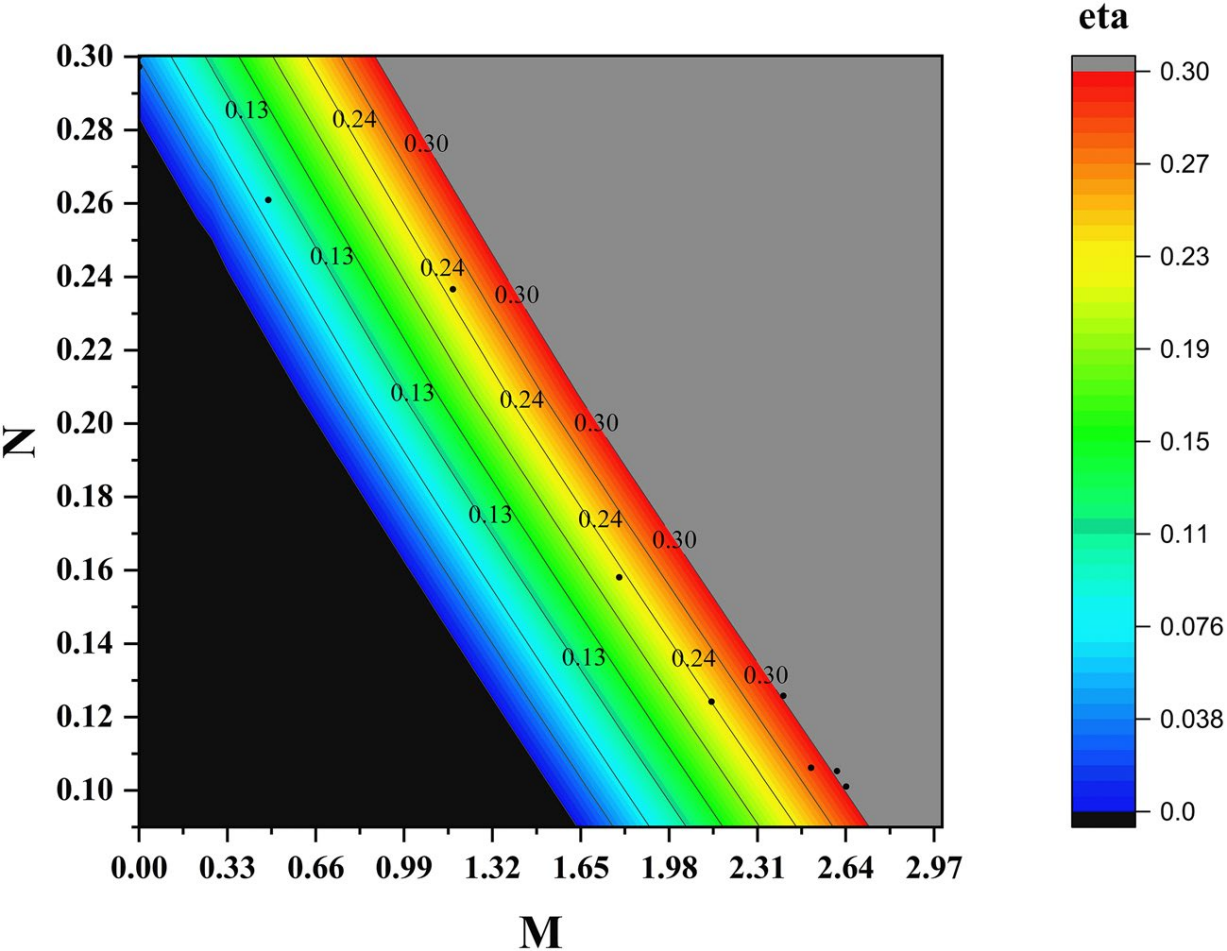
Experimental efficiency compared to pressure ratio N, Entrainment ratio M at 1.45GPM motive flow rate.

At 70% suction closure, the suction pressure notably decreases to  − 0.18 kg/cm^2^, while the entrainment ratio (*M*) only experiences a marginal reduction to 2.41. This is attributed to the slight decrease in suction flow rate from 3.83 to 3.50 GPM, which, despite facing flow resistance, maintains the ability to withstand it. Consequently, this leads to relatively high pressure and entrainment ratios, resulting in a higher efficiency of 30% compared to the fully opened suction valve at 26%. At 40% closure of the suction valve, the suction flow rate experienced a substantial reduction to 1.6 GPM. This was attributed to the elevated flow resistance, resulting in a markedly low entrainment ratio, and consequently contributing to diminished experimental efficiency. (Fig. 5) illustrates the relationship between pressure (*N*) and entrainment (*M*) ratios versus experimental efficiency.

### Case 2

**Figure 6:**
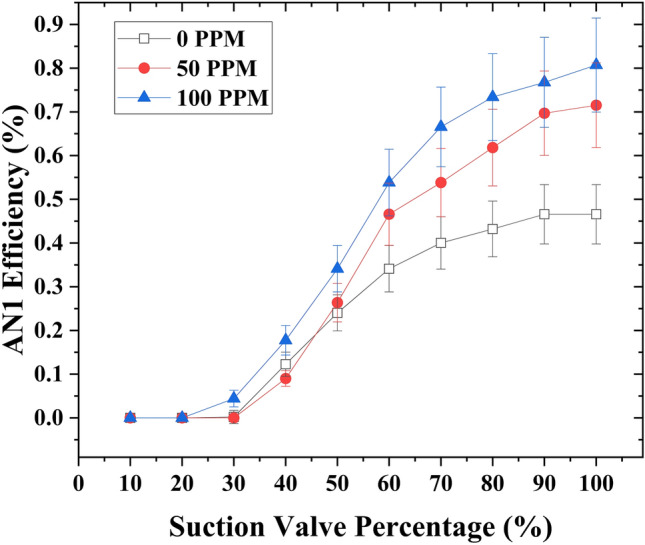
Variation of Analytical type 1 efficiency with respect to the capacity of the suction flow rate control valve at 1 GPM motive flow rate with different DRPs concentrations (0, 50 and 100 ppm).

**Figure 7:**
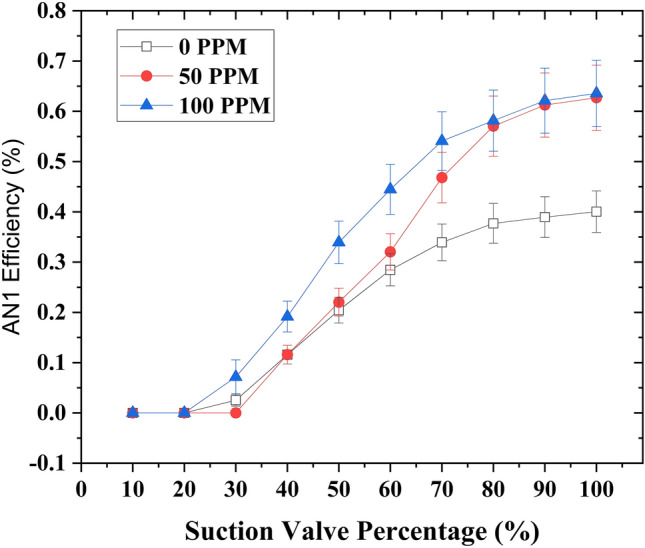
Variation of Analytical type 1 efficiency with respect to the capacity of the suction flow rate control valve at 1.45 GPM motive flow rate with different DRPs concentrations (0, 50 and 100 ppm).

The impact of drag-reducing polymers (DRPs) on analytical type 1 efficiency (*η*_*A*1_) was investigated at a motive flow rate of 1 and 1.45 GPM. (Fig. 6) illustrates the results obtained from various experimental scenarios at a 1 GPM motive flow rate. The addition of DRPs at concentrations of 100 ppm and 50 ppm resulted in higher η_A1_ values. The highest analytical type 1 efficiency of 80.7% was achieved at a suction flow rate of 3.01 GPM with a concentration of 100 ppm DRPs. Subsequently, the DRPs concentration was reduced to 50 ppm, which led to an improvement in the efficiency of Analytical Type 1 compared to the suction flow rate without any DRPs (0 ppm concentration). The analytical type 1 efficiency increased from 46.5% at a suction flow rate of 2.2 GPM to 71% at a suction flow rate of 2.81 GPM. It is worth noting that the efficiency slope decreased noticeably at a concentration of 100 ppm DRPs compared to 50 ppm. Additionally, examining the relationship between suction flow rate control valve capacity and analytical efficiency, as depicted in (Fig. 7), revealed variations observed at different concentrations of DRPs while maintaining a motive flow rate of 1.45 GPM. At 50 ppm, the suction flow rate increased from 2.9 GPM to 3.8 GPM, resulting in an improvement in analytical type 1 efficiency from 40% to 62.7%. Furthermore, when the concentration of DRPs was increased to 100 ppm, the suction flow rate reached 3.83 GPM, with an analytical type 1 efficiency of 63.7%. Comparing the analytical type 1 efficiencies at both DRP concentrations (50 ppm and 100 ppm), similar results were observed, with an approximate 23% enhancement in the system's analytical type 1 efficiency compared to the system with no DRPs addition (0 ppm concentration).

### Case 3

**Figure 8:**
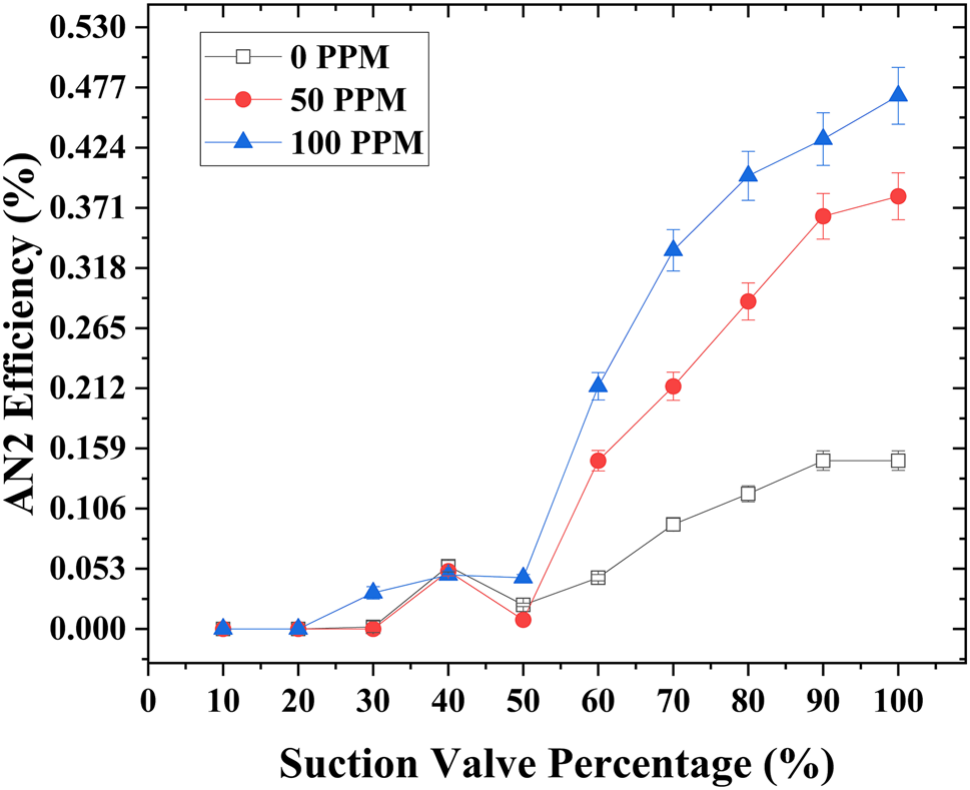
Analytical type 2 efficiency variation as a function of suction flow rate control valve capacity at a motive flow rate of 1 GPM, considering different concentrations of drag reducing polymers (DRPs) (0, 50, and 100 ppm).

The variation of analytical type 2 efficiency (*η*_*A*2_) with the capacity of the suction flow rate control valve was examined using (Fig. 8). The analysis using both figures was conducted at different concentrations of drag-reducing polymers (DRPs) (0, 50, and 100 ppm) while maintaining a motive flow rate of 1 GPM. Introducing DRPs at a concentration of 50 ppm in the suction line resulted in a significant enhancement of the analytical type 2 efficiency (*η*_*A*2_), increasing it from 13 to 38%. This improvement corresponded to an increase in the suction flow rate capacity from 2.2 to 2.81 GPM. Further increasing the concentration of DRPs to 100 ppm led to a substantial improvement in the analytical type 2 efficiency (*η*_*A*2_), rising from 13 to 47%. This enhancement was accompanied by an increase in the suction flow rate capacity to 3.01 GPM. Furthermore, it was observed that the slope of the analytical type 2 efficiency (*η*_*A*2_) appeared to be lower at a concentration of 100 ppm compared to 50 ppm. Additionally, at a lower suction flow rate control valve opening of 40%, the analytical type 2 efficiency yielded nearly identical results across the three different DRP concentrations (0, 50, and 100 ppm), with higher values compared to the 50% suction control valve opening. Furthermore, the analytical type 2 efficiency remained consistent across the three DRP concentrations (0, 50, and 100 ppm) at this lower valve opening; furthermore, the analytical type 2 efficiency (*η*_*A*2_) demonstrated a higher value at a 40% opening of the suction control valve compared to a 50% opening of the suction flow rate control valve.

**Figure 9:**
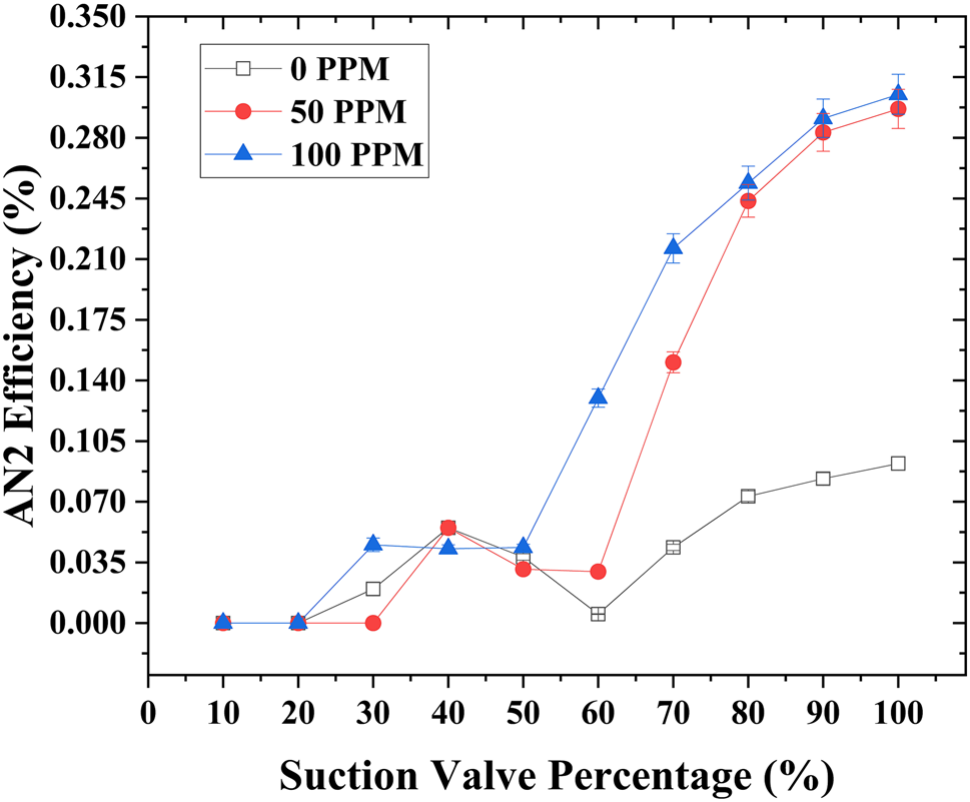
Analytical type 2 efficiency variation as a function of suction flow rate control valve capacity at a motive flow rate of 1 .45 GPM, considering different concentrations of drag reducing polymers (DRPs) (0, 50, and 100 ppm).

Moreover, under a constant motive flow rate of 1.45 GPM, the introduction of drag-reducing polymers (DRPs) at a concentration of 50 ppm into the suction line yielded a noteworthy enhancement in the analytical type 2 efficiency (*η*_*A*2_). It went from 8% to 27.7%, resulting in an increased suction flow rate capacity from 2.9 to 3.8 GPM. Elevating the DRPs concentration to 100 ppm exhibited a similar improvement in the analytical type 2 efficiency (*η*_*A*2_), which increased from 8% to 28.5%. The suction flow rate capacity also increased, reaching 3.83 GPM. Importantly, the slope of the analytical type 2 efficiency (*η*_*A*2_) remained consistent between 100 and 50 ppm DRPs concentrations. Furthermore, at a lower suction flow rate control valve opening of 40%, the analytical type 2 efficiency showed higher values at a concentration of 50 ppm compared to 100 ppm. Additionally, it exhibited higher efficiency (*η*_*A*2_) than at a 50% suction flow rate control valve opening. Moreover, the same behavior of 1 GPM is observed here; at a 40% lower valve opening, the efficiency (*η*_*A*2_) was higher compared to when the suction flow rate control valve opening was set at 50%. The relationship between the capacity of the suction flow rate control valve and the analytical type 2 efficiency (*η*_*A*2_) is illustrated in (Fig. 9).

### Case4

**Figure 10:**
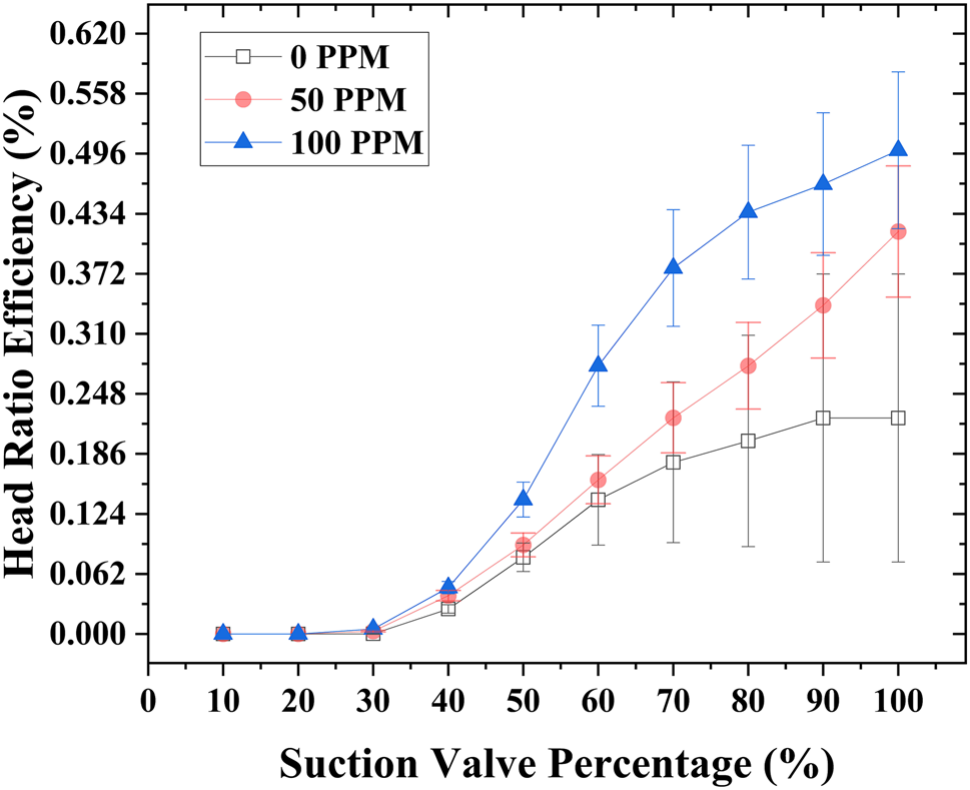
Variation of head ratio efficiency with respect to the capacity of the suction flow rate control valve at 1 GPM motive flow rate with different DRPs concentrations (0, 50 and 100 ppm).

The application of drag-reducing polymers (DRPs) at a concentration of 100 ppm has demonstrated a significant enhancement in the head ratio efficiency (*η*_*HR*_) at a motive flow rate of 1 gallon per minute (GPM), raising it from 22.3% to 50%. Additionally, when the DRP concentration was reduced to 50 ppm, the head ratio efficiency exhibited a noticeable improvement, reaching 41.5%. Notably, the slope of the head ratio efficiency is lower at 100 ppm DRPs concentration compared to 50 ppm, indicating a higher conservation of head ratio efficiency at higher suction flow rate control valve openings. Furthermore, reducing the opening of the suction flow rate control valve did not lead to an increase in head ratio efficiency when compared to the analytical or experimental efficiencies. (Fig. 10) provides a visual representation of the observed variations.

**Figure 11:**
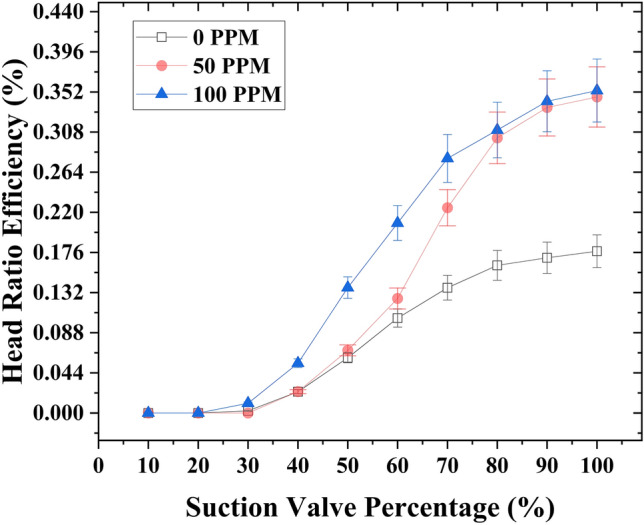
Variation of head ratio efficiency with respect to the capacity of the suction flow rate control valve at 1.45 GPM motive flow rate with different DRPs concentrations (0, 50 and 100 ppm).

A significant improvement in head ratio efficiency is observed with the introduction of a 100-ppm concentration of drag-reducing polymers (DRPs) at a motive flow rate of 1.45 GPM, resulting in an increase from 17.7% to 35.3%. Interestingly, the effects of 100 ppm and 50 ppm concentrations on the capacity of the high-suction flow rate control valve openings appear to be similar. However, when the suction flow valve is closed, the efficiency of the 100-ppm concentration surpasses that of the 50-ppm concentration. Furthermore, when the valve opening is significantly reduced, both the 100 ppm and 50 ppm concentrations show comparable improvements in head ratio efficiency compared to the suction flow rate without DRPs (0 ppm). Observations are depicted in (Fig. 11).

### Cases efficiencies comparison

A comparison was conducted between the efficiencies of different cases at two motive flow rates (1 and 1.45 GPM), with the suction flow rate control valve fully open. It was observed that at a motive flow rate of 1.45 GPM, the head ratio, analytical type 2, and experimental efficiencies showed relatively close results, whereas the analytical type 1 efficiency differed. However, at a motive flow rate of 1 GPM, the experimental efficiency deviated from both the head ratio and analytical type 2 efficiencies. This deviation can be attributed to the models' dependence on the entrainment ratio (*M*), which tends to yield higher values at low motive flow rates, thereby increasing the efficiency of the system according to these models. The head ratio efficiency model appears to closely align with the experimental results; however, when incorporating loss coefficients, it compensates for the deficit observed in experimental efficiency, thereby making it the closest approximation to the actual efficiency state. Table 5 effectively illustrates the comprehensive comparison. The absolute uncertainty of the full scale of the motive and suction flowrate and motive, suction and delivery pressure are shown in Table 6.

**Table 5:** Comprehensive comparison between the different cases efficiencies at different motive flow rates (1 and 1.45 GPM) and DRPs concentrations (0, 50 and 100 ppm) at fully opened suction control valve.

Motive flow rate (GPM)	Concentration (PPM)	*SVC* (%)	*η*_*EX*_ (%)	*η*_*AN*1_ (%)	*η*_*AN*2_ (%)	*η*_*HR*_ (%)
1.45	100	100	26.69	63.58	30.51	35.3
1.45	50	100	25.33	62.7	29.67	34.59
1.45	0	100	13.82	40.03	9.203	17.71
1	100	100	30.99	80.72	46.97	49.86
1	50	100	24.61	71.07	37.67	41.49
1	0	100	6.56	46.58	14.83	22.29

**Table 6:** Uncertainty of the full scale.

	*Q*_*m*_ (GPM)	*Q*_*s*_ (GPM)	*P*_*m*_ (kg/cm^2^)	*P*_*s*_ (kg/cm^2^)	*P*_*d*_ (kg/cm^2^)
Uncertainty	0.04	0.04	0.01	0.04	0.002

## Conclusion

To enhance the performance and efficiency of the liquid ejector, a series of Water-Water experiments were conducted without modifying the ejector's design specifications. The experiments involved the addition of drag-reducing polymers (DRPs) at varying concentrations, specifically 50 and 100 ppm. The following are the conclusions of our study:

1. Optimal performance was observed at a motive flow rate of 1 GPM with the addition of 100 ppm DRPs in the suction stream.
2. At a motive flow rate of 1.45 GPM, a notable improvement in efficiency was observed when the suction valve was partially opened to 70%, contrasting with the fully opened configuration in the experimental efficiency model (Case 1). This modification resulted in an efficiency increase from 26 to 30%. Conversely, in the analytical efficiency models, controlling the suction flow rate valve did not yield any enhancement in efficiency.
3. Identified an inverse relationship between entrainment ratio and pressure ratio; as the motive flow rate increased, pressure ratio increased while entrainment ratio decreased.
4. To achieve the maximum level of efficiency, finding an optimal point in the liquid jet pump requires finding a balance between entrainment and pressure ratios.

## Data Availability

All data generated or analyzed during this study are included in this published article. The corresponding author can be contacted if there is a request for the data.

## List of symbols

*A*_*N*_: Nozzle area, M^0^L^2^T^0^ (m^2^)
*As*: Suction pipe area, M^0^L^2^T^0^ (m^2^)
${A}_{ME}$: Mixing chamber area ratio, M^0^L^0^T^0^
*C*: Density ratio, M^0^L^0^T^0^
*DR*: Drag reduction, M^0^L^0^T^0^
$${D}_{n}$$: Nozzle diameter, L (m)
$${D}_{t}$$: Throat diameter, L (m)
${H}_{m}$: Motive/primary flow head, L (m)
${H}_{s}$: Suction flow head, L (m)
${H}_{d}$: Discharge flow head, L (m)
${K}_{p}$: Primary flow loss coefficient, M^0^L^0^T^0^
${K}_{s}$: Suction flow loss coefficient, M^0^L^0^T^0^
${K}_{m}$: Mixing chamber flow loss coefficient, M^0^L^0^T^0^
${K}_{d}$: Discharge flow loss coefficient, M^0^L^0^T^0^
${K}_{f,m}$: Primary flow friction loss coefficient, M^0^L^0^T^0^
${K}_{f,s}$: Suction flow friction loss coefficient, M^0^L^0^T^0^
${K}_{f.d}$: Discharge flow friction loss coefficient, M^0^L^0^T^0^
*M*: Entrainment ratio, M^0^L^0^T^0^
*N*: Pressure ratio, M^0^L^0^T^0^
${P}_{d}$: Discharge pressure, ML^−^^1^ T^−^^2^ (kg/cm^2^)
${P}_{s}$/*SP*: Suction pressure, ML^−^^1^ T^−^^2^ (kg/cm^2^)
${P}_{m}$: Motive pressure, ML^−^^1^ T^−^^2^ (kg/cm^2^)
${Q}_{d}$: Discharge flow rate, M^0^L^3^T^−^^1^ (GPM)
${Q}_{p}$: Primary/motive flow rate, M^0^L^3^T^−^^1^ (GPM)
${Q}_{s}$/*SFR*: Suction flow rate, M^0^L^3^T^−^^1^ (GPM)
*R*: Area ratio
*SVC*: Suction valve capacity
${V}_{d}$: Discharge flow velocity, LT^−^^1^ (m/s)
${V}_{m}$: Primary/motive flow velocity, LT^−^^1^ (m/s)
${V}_{n}$: Nozzle flow velocity, LT^−^^1^ (m/s)
${V}_{s}$: Suction flow velocity, LT^−^^1^ (m/s)
*X*: Flow rate fraction
$\Delta {P}_{with DRA}$: Pressure drop with drag reducing agent, ML^−^^1^ T^−^^2^ (kg/cm^2^)
$\Delta {P}_{without DRA}$: Pressure drop without drag reducing agent, ML^−^^1^ T^−^^2^ (kg/cm^2^)
${\eta }_{A1}$: Analytical type 1 efficiency, M^0^L^0^T^0^
${\eta }_{A2}$: Analytical type 2 efficiency, M^0^L^0^T^0^
$${\eta }_{EX}$$: Experimental efficiency, M^0^L^0^T^0^
${\eta }_{HR}$: Head ratio efficiency, M^0^L^0^T^0^
${\Theta }_{d}$: Divergence angle, M^0^L^0^T^0^ (deg)
${\rho }_{p}$: Primary fluid density, ML^−^^3^ (kg/m^3^)
${\rho }_{s}$: Secondary fluid density, ML^−^^3^ (kg/m^3^)
